# A two-stage renal disease classification based on transfer learning with hyperparameters optimization

**DOI:** 10.3389/fmed.2023.1106717

**Published:** 2023-04-05

**Authors:** Mahmoud Badawy, Abdulqader M. Almars, Hossam Magdy Balaha, Mohamed Shehata, Mohammed Qaraad, Mostafa Elhosseini

**Affiliations:** ^1^Department of Computers and Control Systems Engineering, Faculty of Engineering, Mansoura University, Mansoura, Egypt; ^2^Department of Computer Science and Informatics, Applied College, Taibah University, Al Madinah Al Munawwarah, Saudi Arabia; ^3^College of Computer Science and Engineering, Taibah University, Yanbu, Saudi Arabia; ^4^Department of Bioengineering, Speed School of Engineering, University of Louisville, Louisville, KY, United States; ^5^Department of Computer Science and Engineering, Speed School of Engineering, University of Louisville, Louisville, KY, United States; ^6^Department of Computer Science, Faculty of Science, Amran University, Amran, Yemen; ^7^TIMS, Faculty of Science, Abdelmalek Essaadi University, Tetouan, Morocco

**Keywords:** renal diseases (RD), AI-based diagnosis, convolutional neural network (CNN), metaheuristic optimization, Sparrow Search Algorithm (SpaSA), transfer learning (TL)

## Abstract

Renal diseases are common health problems that affect millions of people around the world. Among these diseases, kidney stones, which affect anywhere from 1 to 15% of the global population and thus; considered one of the leading causes of chronic kidney diseases (CKD). In addition to kidney stones, renal cancer is the tenth most prevalent type of cancer, accounting for 2.5% of all cancers. Artificial intelligence (AI) in medical systems can assist radiologists and other healthcare professionals in diagnosing different renal diseases (RD) with high reliability. This study proposes an AI-based transfer learning framework to detect RD at an early stage. The framework presented on CT scans and images from microscopic histopathological examinations will help automatically and accurately classify patients with RD using convolutional neural network (CNN), pre-trained models, and an optimization algorithm on images. This study used the pre-trained CNN models VGG16, VGG19, Xception, DenseNet201, MobileNet, MobileNetV2, MobileNetV3Large, and NASNetMobile. In addition, the Sparrow search algorithm (SpaSA) is used to enhance the pre-trained model's performance using the best configuration. Two datasets were used, the first dataset are four classes: cyst, normal, stone, and tumor. In case of the latter, there are five categories within the second dataset that relate to the severity of the tumor: Grade 0, Grade 1, Grade 2, Grade 3, and Grade 4. DenseNet201 and MobileNet pre-trained models are the best for the four-classes dataset compared to others. Besides, the SGD Nesterov parameters optimizer is recommended by three models, while two models only recommend AdaGrad and AdaMax. Among the pre-trained models for the five-class dataset, DenseNet201 and Xception are the best. Experimental results prove the superiority of the proposed framework over other state-of-the-art classification models. The proposed framework records an accuracy of 99.98% (four classes) and 100% (five classes).

## 1. Introduction

Kidney stones are one of the most common contributing factors to kidney function loss and, if left untreated, can lead to chronic kidney disease (CKD) development (1). Kidney stones are a common health problem that affects 1–15% of the world's population and is becoming more common with each passing year (2). For example, every year, over two million people in the USA seek treatment at an emergency department for renal colic or stone-related back pain (3). In addition, around two million patients worldwide are in the kidney replacement stage (4).

Kidney stones cause various abnormalities, such as renal failure, loss of employment due to extreme pain, and decreased life quality due to urinary system obstruction. Kidney stones disease occurs due to the accumulation of salt and mineral crystals that are excreted in the urine and turn into stones. Kidney stones develop due to a lack of regular activity and poor dietary habits. Furthermore, chronic conditions such as high blood pressure, diabetes, and obesity can impact stone development. After treatment, the kidney stone may reoccur and become chronic. Kidney function impairment due to the formation of kidney stones endangers human life. Therefore, preventing kidney stone formation and recurrence is still a significant problem for human health (2).

**Figure 1:**
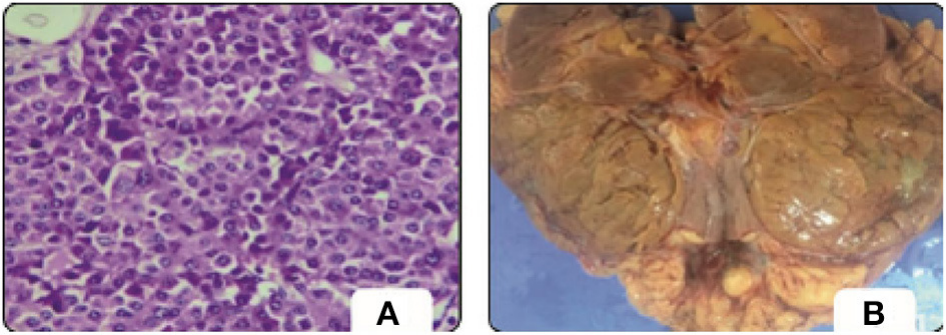
Oncocytoma. **(A)** Microscopy. **(B)** Cut surface (5).

Meanwhile, renal tumors are a major cause of morbidity around the world. Renal cancer is the tenth most prevalent type of cancer, accounting for 2.5% percent of all cancers, according to Modepalli et al. (5). Renal cell carcinoma (RCC) is the most frequent type of kidney cancer, accounting for around 2% of cancer-related mortality worldwide (6). In the USA, kidney cancer is expected to cause 73,750 new cases and 14,830 deaths in 2020 (7). Figure 1 (5) shows the renal Oncocytoma Microscopy and Cut surface. The WHO classification of renal tumors distinguishes 12 different RCC subtypes. Clear-cell RCC (ccRCC) accounts for around 80% of all RCC cases (7). As a result, surgeons must look for new microscopic findings in RCC diagnoses and classification (5). Therefore, renal tumor histological reclassification is critical based on molecular, clinical, and pathological features. Experienced pathologists are required to diagnose RCC using microscopic histopathology slides. The routine histopathological evaluation for a very small amount of tissue is time-consuming and labor-intensive due to the complication of renal neoplasms (8). Besides, some cases are difficult to diagnose and require additional immunohistochemistry testing. Pathological diagnosis of RCC requires novel low-cost, and efficient approaches.

Ultrasonography (USG), magnetic resonance imaging (MRI), and Computed tomography (CT) are the common renal imaging modalities. The clinical condition determines the appropriate Renal imaging technique, the clinical goal, and patient-specific factors such as intensity inhomogeneity within the kidney, the spatial localization of the kidney, the shape variability, and certain congenital anomalies (1).

Ultrasound (US) imaging is a commonly used radio-free diagnostic tool that assesses the size and morphology of the kidneys. Cysts, stones, and tumors can all be detected in the US. It provides good anatomical detail as well as real-time examination. On the other hand, the operator's experience is crucial in image acquisition. The US imaging may be interpreted differently by the radiologists (9). In addition, speckle noise can be seen in the low image quality (1).

Furthermore, multiple US images of the same kidney may appear differently (10). MRI provides high spatial resolution and anatomical and functional information on renal. MRI imaging can also detect renal abnormalities and malignancies. Recently, Advanced MRI techniques have gained considerable attention, such as dynamic contrast-enhanced (DCE) MRI, Blood oxygen level-dependent (BOLD) MRI, and Diffusion-weighted (DW) MRI. However, MRI cannot identify classifications, namely renal stones (1). CT provides information similar to the US but in higher spatial resolution and sensitivity. CT provides clearer vascular tomographic images that depict functions and properties to distinguish interior design elements such as size, density, and structure. In addition, it provides a high-precision evaluation of masses, kidney injuries, and stones. Thus, CT is Effective in diagnosing post-transplant complications. The biggest disadvantage is that it exposes patients to ionizing radiation. Table 1 summarizes the common Renal imaging modalities.

**Table 1:** Common renal imaging modalities.

**Technique**	**Pros**.	**Cons**.
US	• Commonly used in nephrology. • Low cost. • Safe. • Easily operated. • Real-time examination.	• Low contrast. • Artifacts. • Low signal-to-noise ratio. • Hampering the segmentation process.
CT	• High spatial resolution. • High-precision evaluation. • Low specificity.	• The use of contrast agents continues to cause nephrotoxicity. • Exposure to X-ray radiation.
DCE MRI	• Excellent anatomical and functional knowledge.	• Affect the kidney. • Cause nephrogenic systemic fibrosis.
BOLD MRI	• Renal oxygenation status evaluation.	• Breathing motion artifacts are a risk.
DW MRI	• Ability to assess whole kidney perfusion and diffusion. • It detects the movement of water molecules within the tissue.	• Limited to respiratory motion artifacts. • Issues related to protocol variability.

The textural analysis is a useful adjunct that quantifies medical images by analyzing image pixels. It is based on mathematical techniques, investigates the spatial arrangement of gray-level pixels, and reveals relationships. Textures can represent histological variability due to renal architecture and the influence of renal disease on the distribution of functional indicators (11). Combining textural analysis and classic machine learning approaches broadens medical imaging potential in diagnosing and predicting renal dysfunction indistinguishable from the radiologist's eye. The process involves image acquisition, feature extraction, feature selection, segmentation, and classification. Although the early diagnosis of RD patients has been linked to high medical costs and mortality savings, referrals are frequently made too late in the disease course (12). On the other hand, early detection of RD results in better patient management and a lower mortality rate by preventing progression to the endpoint (13). However, the manual procedure is tightly coupled with the experience of nephrologists to diagnose a patient's condition correctly. Besides, it is time-consuming, error-prone, subjective, and inconsistent. Therefore, computer-aided diagnosis (CAD) approaches to aid in preventing progression of RD. CAD's first concern is automating the earlier diagnosis stage based on Artificial Intelligence (AI) techniques. AI significantly impacts healthcare applications, especially in analyzing medical images.

Interestingly, Deep learning (DL), a field of artificial intelligence, has been exceedingly used and made a significant guide in building renal function evaluation frameworks. Thus, a fully accurate automated procedure for early disease diagnosis based on deep learning algorithms plays an essential role in the patient's survival. Furthermore, deep learning has proven to be promising in interpreting medical images that surpass human experts; besides, it minimizes physician-induced errors due to its powerful classification, detection, and segmentation capabilities (14).

### 1.1. Paper contributions

To the best of our knowledge, deploying hyperparameter optimization with automated RD detection is still a vague area, and several challenges and issues have not yet been addressed. Therefore, this study proposes a dualistic RD classification (DRDC) framework based on transfer learning (TL) with hyperparameters optimization. The DRDC framework performs automatic, accurate kidney stones and tumors classification using CT images and microscopic histopathological examination. The DRDC uses an optimized Convolutional Neural Network (CNN) by the Sparrow Search Algorithm (SpaSA) (14, 15). The contributions are as follows:

- Proposing DRDC framework for accurately classifying kidney stones and tumors based on the CT images and microscopic histopathological.
- The SpaSA optimizes the CNN parameters and hyperparameters to improve classification accuracy by finding the optimal configurations for the CNN models.
- The proposed framework is characterized by adaptability via automatic assignment of the CNN architecture's hyperparameters.
- Two distinct datasets were used in the experiments. The first database is classified into four CT classes, Normal, Cyst, Tumor, and Stone, while the second dataset is classified into five histopathological classes, in case of tumor exists.
- The proposed method yields very promising outcomes compared to state-of-the-art techniques,
- A manual error analysis is conducted to determine the reason behind the misclassification and how to rectify it.

### 1.2. Paper organization

This paper is organized as follows: Section 2 summarizes related works about automatic diagnosis of different RD in healthcare informatics. Section 3 introduces the background of computer-aided decision support systems props. Section 4 presents the proposed framework DRDC in detail. In Section 5, the experiments are introduced, and the findings are discussed. The final section summarizes the findings and draws the necessary conclusions.

## 2. Related studies

Yildirim et al. (3) proposed an automated detection and localization technique for kidney stone diagnosis using DL. A binary classifier based on 1,799 CT images is introduced on a four-stage XResNet-50 model. CT images are the input to the model, and the output class is provided beside the region of interest (RoI). The labeling process is carried out by the experts carried out without CT image segmentation. The proposed model obtained a proper diagnosis with an accuracy of 96.82%. However, the model gave erroneous results and focused only on the stomach. In addition, a limited dataset is used, which limits the model's generalizability. Aksakalli et al. (2) developed a DL model that detects whether a kidney X-ray image is patient or healthy. The proposed method comprises six phases: scaling, resizing, gray-level values extraction, generating CSV, resampling, and evaluation. They used small data set of 221 kidney X-ray images. Experiments demonstrated that the proposed method achieved an F1-score with a success rate of 85.3% utilizing the S + U sampling method.

Ma et al. (16) proposed a Heterogeneous, Modified Artificial Neural Network (HMANN) method for the multi-classification of kidney stones. The deep learning-based HMANN performs preprocessing, feature extraction, segmentation, and chronic renal failure classification. Based on an ultrasound image, the model achieves high accuracy of 97.5% in predicting kidney stones and the RoI. However, the HMANN relies on a small CT dataset. An ensemble of pre-trained DNN -based methods are introduced in (4) for kidney stone muti-classification (normal, cyst, stone, and tumor). The method consists of four processes: augmentation, speckle noise, TL of DNNs, and classification process. They used a dataset consisting of 4,940 augmented US images. However, a modification in the architecture of DNNs is needed to improve the accuracy. Then, the authors proposed a kidney stone multi-Classification CAD based on US image diagnosis System (9). They aimed to remove Speckle noise in the US images using a deep residual learning network (RLN). They used a pre-trained ResNet-101 model for Feature extraction and SVM for classification. For training and testing, they used 4,940 augmented US images.

Zheng et al. (17) studied the US anatomic characteristics of children's kidneys as biomarkers of children with congenital abnormalities of the kidney and urinary tract (CAKUT). They used a deep TL approach for developing a binary classification for control and children with CAKUT. However, they used a limited dataset consisting of only 50 patients. An ML-based CAD system that combines imaging markers and clinical biomarkers is developed to detect acute renal transplant rejection (18). The proposed system consists of data prepossessing, ROI selection; 3D map extraction; and classification. Although the proposed system offers high reliability and non-invasive diagnosis, it requires a larger sample size. Patil and Choudhary et al. (19) developed a deep CKD risk classification new prediction model based on US images. The proposed model consists of preprocessing, feature extraction, and classification. Feature extraction involves texture analysis, local binary pattern (LBP) model, area extraction, and mean intensity extraction.

An optimized deep CNN is used with the DM-HWM model's optimization. They used a manually collected dataset that contains 137 US images. The model achieves a Sensitivity of 89.98. However, it depends on the small sample size. Yin et al. (20) developed a multi-instance CNN-based learning classifier based on 2D US images. For the optimization of features learned from CNN, they used GNNs. The proposed method achieves about 85% of accuracy. However, the automated network architecture optimization is still missing. Smail et al. (21) developed a five-layer CNN to classify five-way Grade Hydronephrosis Severity (GHS) based on US images. Deep learning algorithms are used to provide human grading experts. DL standards limited the dataset, and it was collected from 687 patients. Dataset was imbalanced and only contained one image per patient visit. The Detection of Autosomal Dominant Polycystic Kidney Disease (ADPKD) is time-consuming and costly. Besides, tracking the progression of ADPKD disease over time is essential for treatment. Brunetti et al. (22) developed an automated CNN-based procedure to segment and classify ADPKD. They used the Genetic algorithm (GA) for CNN's architecture optimization. They used a limited dataset of 526 MRI images.

Nazari et al. (6) created a machine learning-based model to predict RCC patients' overall survival. The proposed model consists of the acquisition, manual ROI tumor segmentation, preprocessing, feature extraction, and classification. The best classification accuracy (0.98%) was achieved by the XGBoost model trained and validated on 222 RCC-CT images for 70 patients. Furthermore, a reliable DL-based CNN framework for the segmentation of renal biopsy and nephrectomies histologic primitives (RBNHP) is proposed (23). Multiple DL approaches were trained with optimal digital magnification for the computational derivation of histomorphometric features. This clinical decision support used 459 digital renal biopsies from 38 histology laboratories. Chen et al. (7) introduced a computational recognition machine learning model integrated with clinicopathologic factors. The model aimed at automated and accurate diagnosis and survival prediction of clear cell RCC patients based on histopathologic images. A total of 1,107 images of H&E slides from 947 RCC patients were used. Segmentation and feature extraction pipeline *via* CellProfiler is used. High versus low-risk scores were found; however, the median score was used as the cut-off value.

### 2.1. Related studies summarization

Table 2 summarizes the discussed related studies. They are ordered in descending order according to the publication year. As far as the authors know, this study is the first to (1) investigate the role of transfer learning and hyperparameter optimization along with different renal disease. (2) diagnosis of different RD based on the two-phase classification for stones and tumors.

**Table 2:** Related studies summarization.

**Reference**	**Application**	**Approach**	**Dataset**	**Pros**.	**Cons**.	**Best performance**
Patil and Choudhary (19)	CKD risk classification	HWM + Optimized DCNN	137 US images	Computational time	Small sample size	Sensitivity of 89.98
Yildirim et al. (3)	Kidney stone binary classification	XResNet-50 model	1799 CT images	Identify small size stones	Erroneous results-limited dataset	Accuracy of 96.82%
Sudharson et al. (9)	Multi-class kidney abnormalities	ResNet-101- SVM	4,940 augmented US images	Noise reduction	Low accuracy	Accuracy of 87.31%
Nazari et al. (6)	Death Risk prediction within 5Y in RCC patients	XGBoost	222 RCC CT images	Good accuracy	Small dataset	Accuracy of 0.98%
Jayapandian et al. (23)	RBNHP	U-Net models.	459 digital renal biopsies	Six renal histologic primitives classification	Multiple DL networks can improve the results	F-scores: 0.94
Chen et al. (7)	Survival prediction of ccRCC patients	CellProfiler	1,107 images of H&E slides	Large dataset	External validation- excluded patients with difficult diagnosis	AUC of 97.0% and increased accuracy by 6.6%
Aksakalli et al. (2)	Kidney stone binary classification	Decision Tree (DT)	221 X-ray images	Execution time	Limited dataset	Asuccess rate of 85.3%
Ma et al. (16)	Multi-class kidney abnormalities classification	SVM and back propagation algorithms.	400 CT images	Noise reduction - Low computation time.	Limited dataset	High sensitivity, specificities, and accuracy of 97.5%
Smail et al. (21)	GHS- five-way classification problem.	DCNN	2,420 sagittal US	Moderate accuracy	Small and imbalanced dataset	Accuracy of 94%
Sudharson et al. (4)	TKidney stone multi- classification.	ResNet-101, ShuffleNet, and MobileNet-v2	4,940 augmented US images	Noise reduction	Low accuracy	Accuracy of 96.54%
Zheng et al. (17)	CAKUT binary Classification	Imagenet-caffe-Alex	120 US CAKUT-150 US Control	TL and the conventional imaging features integration	Limited dataset - Low accuracy	Accuracy of 87%
Abdeltawab et al. (18)	Detection of acute renal transplant rejection	TReLU	DW-MRI of 56 individuals	A reliable non-invasive diagnosis	Small sample size	Accuracy of 92.9%
Yin et al. (20)	CAKUT binary Classification	CNN +GNNs	2,687 US CAKUT- 2,246 US Control	Large Data sets	Network architecture optimization	Accuracy of 85%
Brunetti et al. (22)	ADPKD	CNN +GA	526 MRI images	Moderate accuracy	Small dataset	Accuracy of 95%

## 3. Background

In recent years, doctors have increasingly used machine learning as a diagnostic tool, which provides them with complementary information (24). Recently, deep learning (DL) has been applied in many medical imaging analysis techniques. Convolution neural networks (CNNs) have been widely used in solving many problems, especially classification (25). CNN is one of the most well-known and influential deep learning models in computer vision, speech recognition, and medical diagnosis (14). Deep CNN architecture named AlexNet was shown to be very effective on highly challenging datasets when applied to the ImageNet LSVRC-2012 competition with purely supervised learning (26, 27). With CNN DL Models, relevant features are extracted using various layers of CNNs followed by fully-connected neural networks. In transfer learning, components of a model developed for one purpose are used to construct a new model for a different task. It seems like a very interesting research area to train Deep Learning models on different datasets and transfer layers between the models for various tasks. The novel model incorporates more training data and upgraded neural layers (28, 29). The concept of meta-learning may contribute to achieving a higher level of reuse in the future.

Sparrow Search Algorithm (SpaSA), a revolutionary metaheuristic algorithm introduced in 2020, is primarily inspired by sparrows' foraging and anti-predation behavior. Based on benchmark functions, SpaSA has a better optimization capability and is more efficient than PSO, GWO, LAPO, and other learning algorithms (30). This is because the sparrow has a small brain capacity but is an intelligent, socially cooperative creature with good memory and a good sense of division of tasks. Therefore, SpaSA has proven to be an incredibly powerful optimization algorithm when inspired by the sparrow population's natural foraging and anti-predator behavior (31).

**Figure 2:**
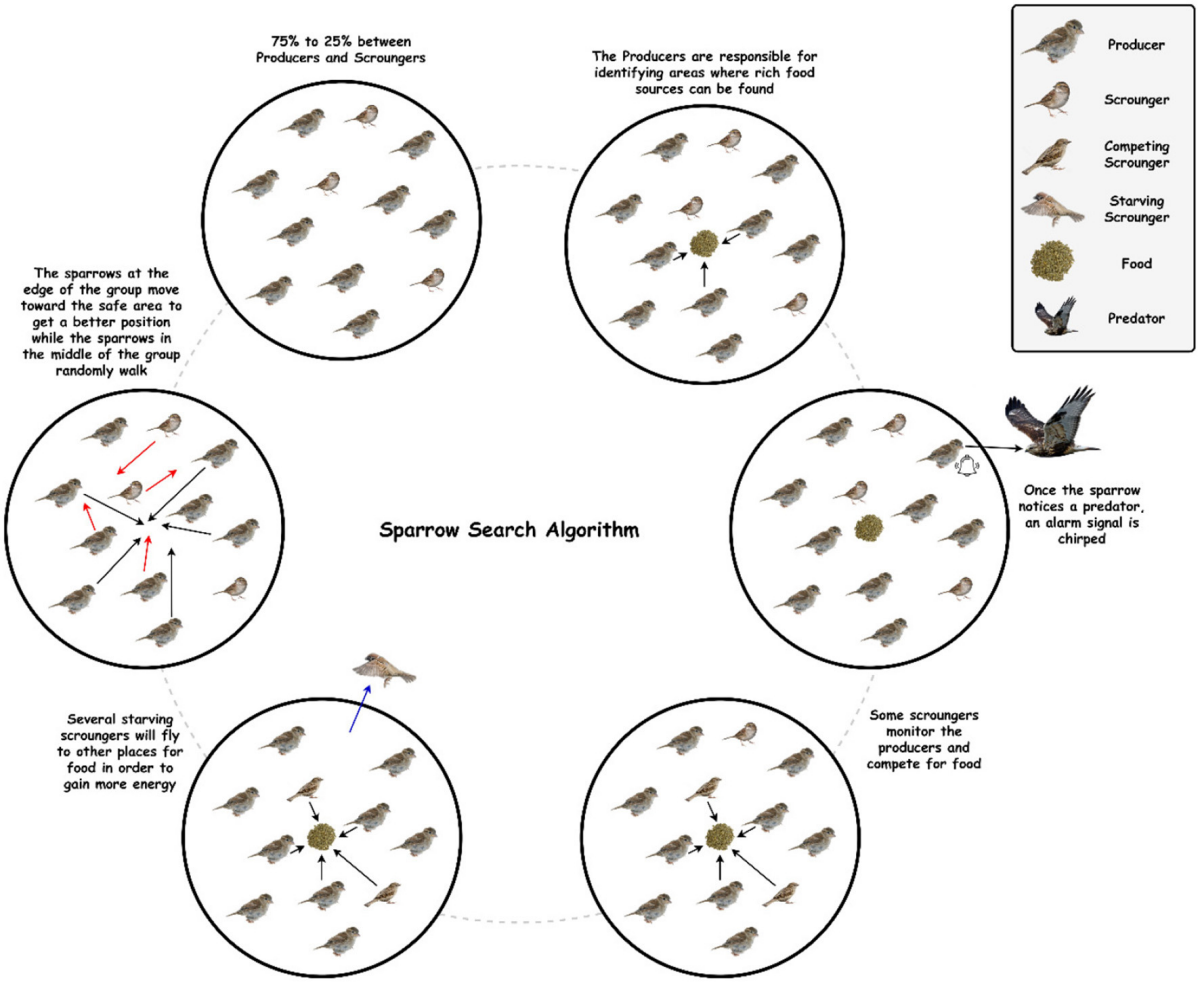
The schematic diagram of sparrow behavior.

In the SpaSA, as shown in Figure 2, sparrow flocks are modeled as they go about foraging. With a superimposed reconnaissance and early warning system, sparrow flocks forage through a discoverer-joiner model. The sparrow population consists of discoverers, joiners, and scouts. As with many animals, individuals adept at finding food tend to be the discoverers, while other individuals play the role of joiners. The discoverer guides the population with a high fitness value, a wide search range, and the ability to guide the population to find food. The joiner follows the discoverer hunting to improve their fitness. Furthermore, a proportion of individuals in the population serve as scouts to watch out for dangers such as predators and companions, thereby improving predation and risk prevention abilities (32, 33).

Six idealized intrinsic rules govern sparrow behavior: (1) The producers maintain high energy reserves and guide all scroungers. (2) When a sparrow discovers a predator, it chirps to alert the other sparrow. (3) In this study, the percentage of producers is set at 20%. On the other hand, each sparrow has the potential to be a producer if it can discover higher quality food supplies and has a larger energy reserve. (4) Scroungers may leave their current places if they become starving. (5) Scroungers stalk those producers who can offer the best food sources. (6) When they detect danger, peripheral sparrows fly toward the center of a group (31, 34, 35).

## 4. Methodology

**Figure 3:**
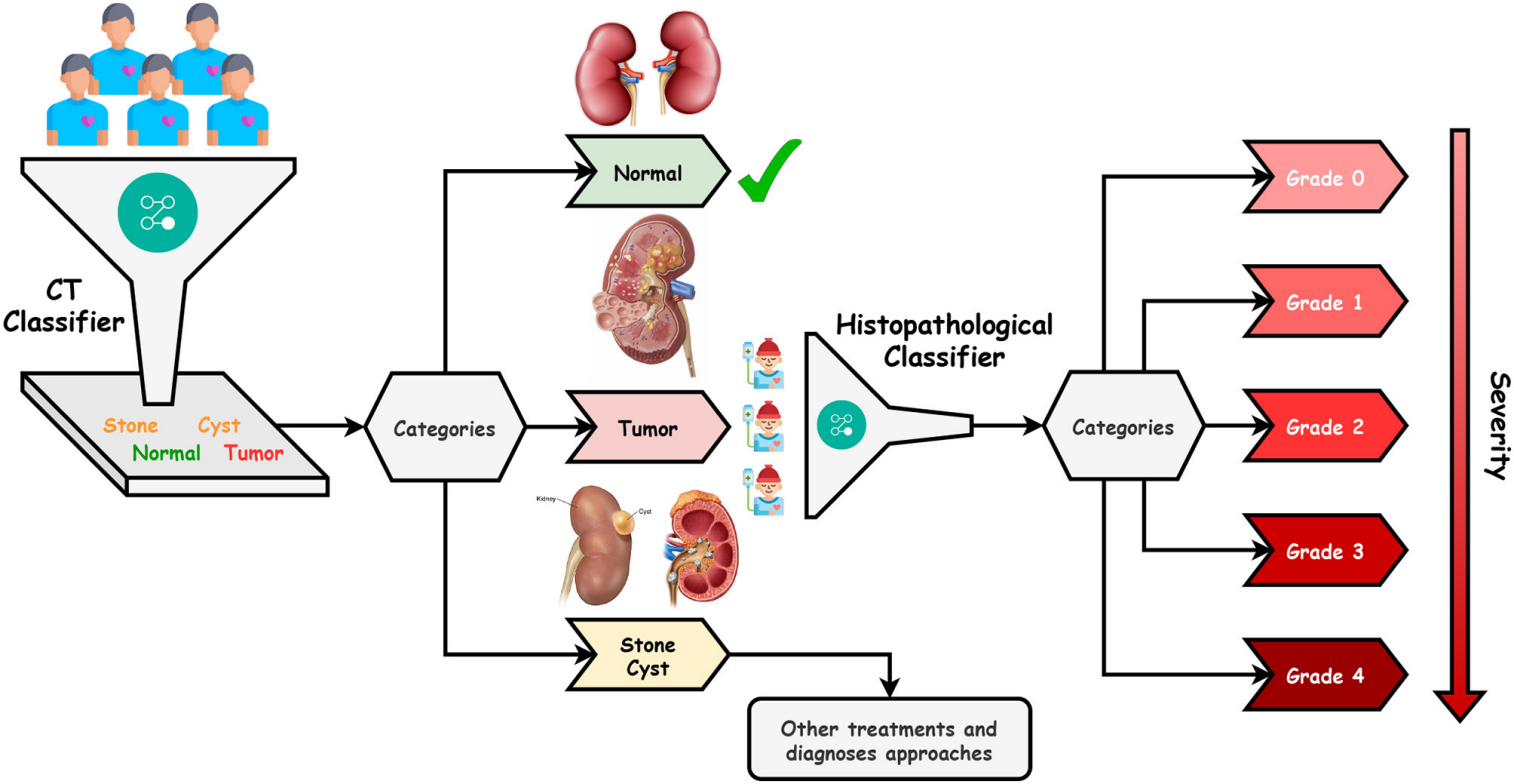
The proposed DRDC framework classification methodology.

**Figure 4:**
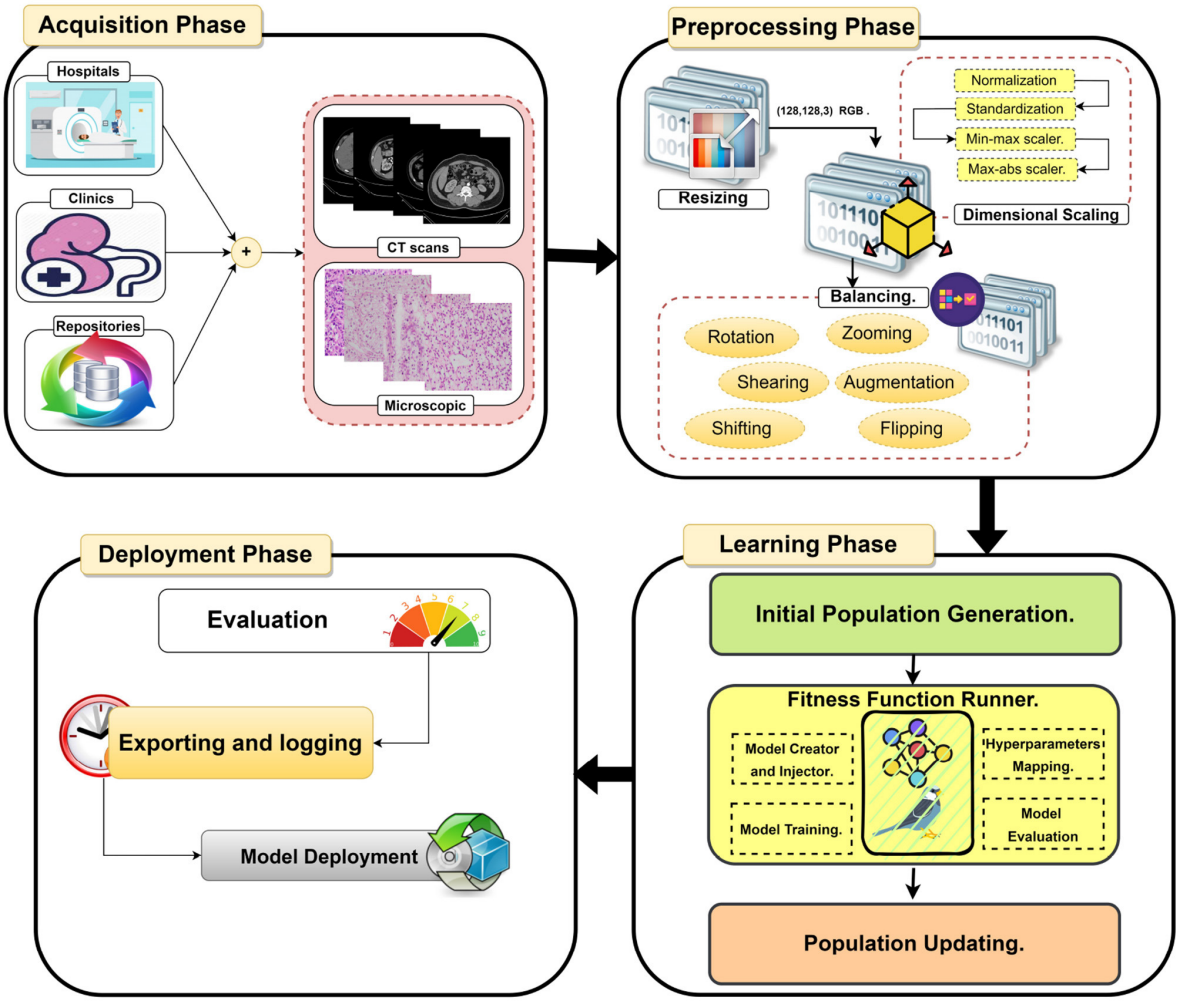
The detailed phases of the proposed DRDC framework.

This paper proposes an efficient Dualistic Renal Disease Classification (DRDC) framework. The DRDC framework is developed for the automatic and accurate classification of the kidney. The DRDC framework uses CT and histopathological kidney images. In addition, the framework aggregates convolution neural networks, transfer learning, and the Sparrow search algorithm. Figure 3 depicts the classification methodology of the proposed DRDC framework. In addition, the DRDC framework phases are detailed as shown in Figure 4.

The patient will first get a CT scan of the kidney, as indicated in Figure 3, and the scan will be labeled with the help of the first recommended classifier. After that, a diagnosis of “Normal,” “Stone,” “Cyst,” or “Tumor” should be assigned. If the scan comes out as “Normal”, the patient's kidneys are fine. If the scan results in a “Tumor”, the patient will undergo microscopic histological analysis to determine the grade of the tumor (or cancer). It is worth noting that “Grade 0” is the lowest while “Grade 4” is the highest. Finally, if the scan is “Stone” or “Cyst”, this patient should follow other treatments and diagnosis approaches.

### 4.1. Phase 1: Dataset acquisition

**Figure 5:**
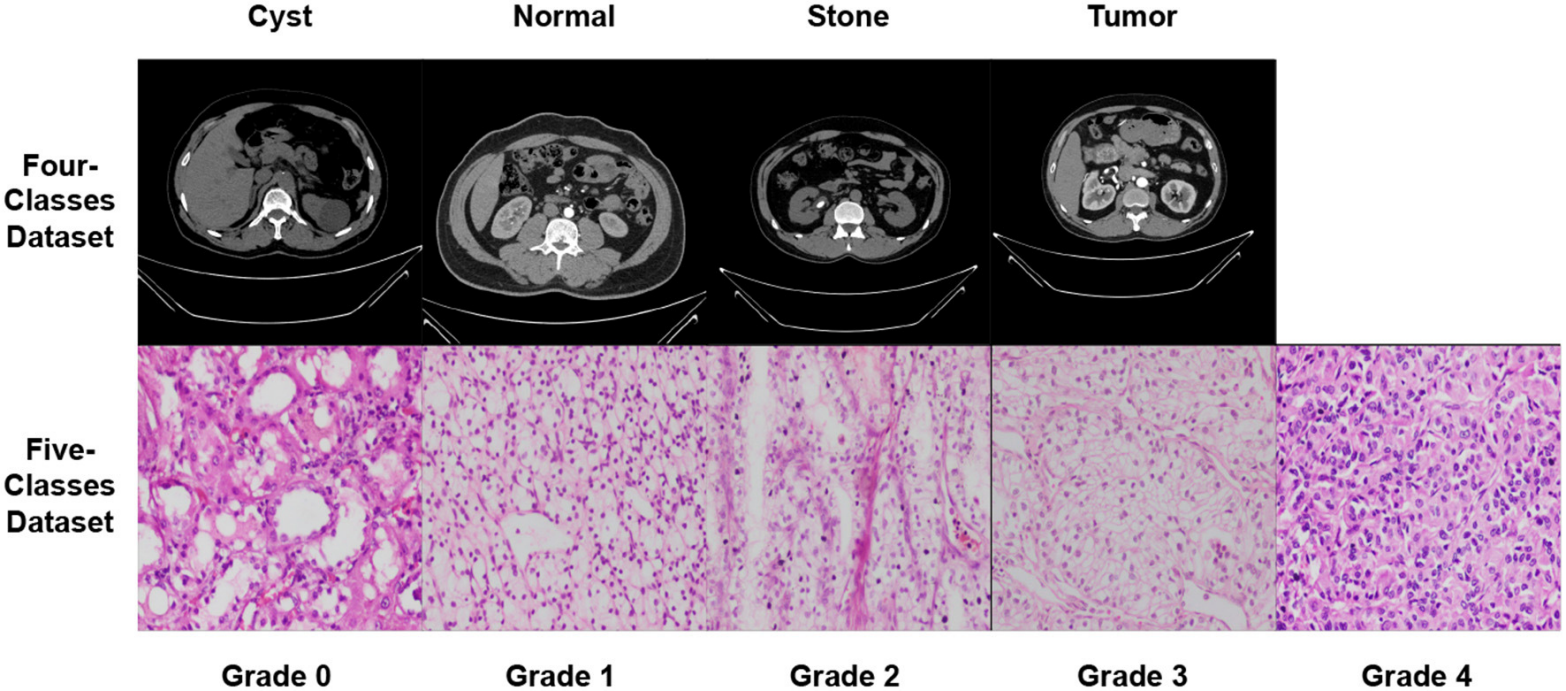
Samples from the used datasets.

Datasets are accessible from several sources, including hospitals, clinics, and online repositories. The datasets used in this study are collected from two public databases (36, 37). The datasets' characteristics are explored in Section 5.1. In summary, this study uses two distinct modalities CT scans and microscopic histopathology examinations (i.e., histopathological slides). Figure 5 depicts images from the used datasets.

### 4.2. Phase 2: Dataset pre-processing

In the second stage, three processes are used to prepare the data sets for further analysis. The processes are resizing, dimensional scaling, and balancing.

#### 4.2.1. Process A: Dataset resizing

The used datasets are not found in the same size; hence, in the RGB color mode, the datasets are shrunk to a size of (128, 128, 3). Finally, the sampling is performed by using bicubic interpolation.

#### 4.2.2. Process B: Dataset scaling

The proposed DRDC framework uses four scaling methods, which will be discussed later. They are (1) normalization $\left(\frac{X}{\max\left(X\right)}\right)$, (2) standardization $\left(\frac{X-\mu }{\sigma }\right)$, (3) min-max scaler $\left(\frac{X-\min\left(X\right)}{\max\left(X\right)-\min\left(X\right)}\right)$, and (4) max-abs scaler $\left(\frac{X}{\left|\max\left(X\right)\right|}\right)$ where *X* is the input image, *X*_*output*_ is the scaled image, μ is the image mean, σ is the image standard deviation.

#### 4.2.3. Process C: Dataset balancing

The used datasets are not found to be balanced. This issue can lead to a high rate of misclassification or overfitting. Therefore, data balancing techniques should be handled; hence, the data augmentation technique is deployed to overcome this issue. The DRDC framework employs rotation, shifts, shearing, zooming, flipping, and brightness augmentation. Table 3 depicts the intended augmentation methods and their related settings employed to provide dataset balancing.

**Table 3:** Different augmentation techniques and the corresponding configurations used to balance the datasets.

**Technique**	**Value**
Rotation	30°
Width shift ratio	20%
Height shift ratio	20%
Shear ratio	20%
Zoom ratio	20%
Brightness change	[0.8:1.2]
Vertical flip	✓
Horizontal flip	✓

### 4.3. Phase 3: Learning phase

After pre-processing the datasets, the learning phase comes in. The current study utilizes the VGG19, DenseNet201, MobileNet, VGG16, NASNetMobile, Xception, MobileNetV3Large, and MobileNetV2 pre-trained CNN models.

In short, VGG16 and VGG19 are CNN models created by the Visual Geometry Group at the University of Oxford. Their architecture is straightforward, utilizing many layers of 3x3 convolutional filters with max pooling layers in between. VGG16 has 16 layers and VGG19 has 19 layers (38). DenseNet201 is another CNN model developed by Huang et al. (39). It employs dense connectivity, allowing each layer to connect to every other layer in a feedforward manner, resulting in optimal parameter utilization and improved feature propagation (39). MobileNet and MobileNetV2 are lightweight CNN models meant for mobile and embedded applications. Depthwise separable convolutions are used, reducing parameters and computations necessary for inference while maintaining high accuracy. MobileNetV3Large is the latest iteration of the MobileNet architecture, incorporating features such as squeeze-and-excitation blocks and hard-swish activation functions. These modifications enhance the accuracy and efficiency of the network (40). Google's NASNetMobile is a CNN model that utilizes neural architecture search (NAS) to discover the optimal architecture for a given task via reinforcement learning. It has demonstrated state-of-the-art accuracy on image classification and object detection tasks (41). Finally, Xception is a CNN model created by Chollet in (42). It employs depthwise separable convolutions in a modified Inception architecture, resulting in reduced parameters and computations necessary for inference while maintaining high accuracy (43).

This phase uses the SpaSA meta-heuristic optimizer for the optimization of hyperparameters (e.g., loss function and batch size). The following mechanism aims to discover the optimum setups for each pre-trained TL model utilized. This phase implements three processes. They are:

− Process A: Initial Population Generation.
− Process B: Fitness Function Runner.
− Process C: Population Updating.

The first process (i.e., Process A) is executed just once, while the other two processes are repeatedly executed for some fixed number of cycles *T*_*max*_.

#### 4.3.1. Process A: Initial population generation

When the learning phase begins, a single random number generation is used to seed the population. A population pack has a maximum of *Nmax* possible solutions. Each solution is a vector sized 1 × *D* where each element is in [0, 1]. Hyperparameters are assumed to be reflected in each solution element. Table 4 shows the solution indexing and the corresponding hyperparameters. We can derive from Table 4 that *D* = 15 if data augmentation is used and *D* = 7 otherwise.

**Table 4:** The solution indexing and the corresponding hyperparameters.

**Element index**	**Corresponding hyperparameter**
1	Loss function
2	Batch size
3	Dropout ratio
4	TL learning ratio
5	Weights (i.e., parameters) optimizer
6	Dimension scaling technique
7	Apply data augmentation or not
8	Rotation value (in case of data augmentation is applied)
9	Width shift value (in case of data augmentation is applied)
10	Height shift value (in case of data augmentation is applied)
11	Shear value (in case of data augmentation is applied)
12	Zoom value (in case of data augmentation is applied)
13	Horizontal flipping flag (in case of data augmentation is applied)
14	Vertical flipping flag (in case of data augmentation is applied)
15	Brightness changing range (in case of data augmentation is applied)

#### 4.3.2. Process B: Fitness function runner

Each solution's fitness function score is calculated in this stage, which contains subprocesses. They are:

− Subprocess B.1: Hyperparameters Mapping.
− Subprocess B.2: Model Creator and Injector.
− Subprocess B.3: Model Training.
− Subprocess B.4: Model Evaluation.

**Subprocess B.1: Hyperparameters mapping**: This subprocess converts the solution in “Process A” to the corresponding hyperparameters as defined in Table 4. **How does this happen?** Assume that you must transform the solution's batch size (the second element) into a hyperparameter. The batch size selection range needs to be established initially. This study uses the “4 → 48 (step = 4)” range. Hence, we have 12 possibilities. We can determine which possibility with a simple calculation (*solution*[*index*] × *length*(*ranges*[*index*])). If the random numeric value is 0.85 and we have 12 possibilities, then the index is 11 (i.e., the batch size value of 44). It is worth noting that each hyperparameter's ranges are defined in Table 6.

**Subprocess B.2: Model creation and injection**: After mapping each element in the solution to the relevant hyperparameter, the target pre-trained TL model will be built with the hyperparameters. The pre-trained TL models employed in the current work are SeresNext50, SeresNext101, SeNet154, MobileNet, MobileNetV2, MobileNetV3Small, and MobileNetV3Large, using the “ImageNet” pre-trained weights.

**Subprocess B.3: Model training**: The pre-trained TL model will start the training for several epochs defined by 5 in this study.

**Subprocess B.4: Model evaluation**: The entire dataset is used to evaluate the pre-trained TL model to validate its generalization. To judge the model performance, different performance metrics are used, such as accuracy $\left(\frac{TP+TN}{TP+TN+FP+FN}\right)$, precision $\left(\frac{TP}{TP+FP}\right)$, specificity $\left(\frac{TN}{TN+FP}\right)$, recall (i.e., sensitivity) $\left(\frac{TP}{TP+FN}\right)$, F1-score $\left(\frac{2\times TP}{2\times TP+FP+FN}\right)$, AUC, IoU, and cosine similarity.

#### 4.3.3. Process C: Population updating

The population is arranged descending by fitness score, with the best solution at the top and the worst at the bottom. This is important to determine $X_{best}^{t}$ and $X_{worst}^{t}$ used in the rest of the process. The SpaSA equations are utilized in this process to update the population. First, the discoverer location update procedure is represented in Equation 1. Next, Equation 2 explains the followers' location updating process. Finally, Equation 4 describes the anti-predation behavior.


(1) $$X^{t+1}=\{\begin{array}{ll} X^{t}\times exp^{\left(\frac{-h}{\alpha \times T_{max}}\right)}, & \text{if }(R_{2}<ST)\\ X^{t}+Q\times L, & \text{Otherwise} \end{array}$$

From Equation 1, *X*^*t*^ is the solution at iteration *t*, *t* is the current iteration number, α is a random number ∈[0, 1], *Q* is a normal distributed random number. *L* represents a 1 × *D* matrix of ones, *R*_2_ and *ST* are the warning and safety values respectively, *R*2∈[0, 1], and *ST*∈[0.5, 1].


(2) $$X^{t}=\{\begin{array}{ll} Q\times exp^{\left(\frac{X_{worst}^{t}-X^{t}}{i^{2}}\right)}, & \text{if }(i>\frac{n}{2})\\ X_{P}^{t}+|X^{t}-X_{P}^{t+1}|\times A^{+}\times L, & \text{Otherwise} \end{array}$$

From Equation 2, $X_{P}^{t}$ is the best position of the discoverer at iteration *t*, $X_{worst}^{t}$ is the iteration's *t* poorest position, *A* is a (1 × *D*) matrix, and *A*^+^ is defined in Equation 3.


(3) $$\begin{array}{l} A^{+}=A^{T}\times {\left(A\times A^{T}\right)}^{-1} \end{array}$$

(4) $$\begin{array}{l} X^{t+1}=\{\begin{array}{cc} X_{best}^{t}+\beta \times |X^{t}-X_{best}^{t}|, & \text{if}\left(f_{i}\neq f_{g}\right)\\ X^{t}+K\times \left(\frac{\left|X^{t}-X_{worst}^{t}\right|}{\left(f_{i}-f_{w}\right)+\epsilon }\right), & \text{Otherwise} \end{array} \end{array}$$

From Equation 4, $X_{best}^{t}$ is the best solution at iteration *t*. β is the control step-size parameter. It is a normal distributed random number, *K* is a random number ∈[−1, 1] and it depicts the movement direction and the sparrow, as well as controlling the moving step size, *f*_*i*_ denotes the current sparrow individual fitness value, *f*_*g*_ and *f*_*w*_ are the optimal and worst fitness values respectively, and ϵ is a very small floating-point number to avoid the division by zeros.

Algorithm 1 explains the SpaSA meta-heuristic optimizer population (i.e., solution) updating process.

**Algorithm 1:** The population (i.e., solutions) updating process using the SpaSA meta-heuristic optimizer.

**1 Function Update SpaSA Solutions** (*solutions, scoresList*) // Sort the population scores.
**2 Sort SpaSA Solutions** (*solutions, scoresList*) ; // Sort the scores list in descending order.
3 *best, worst, optimal, bestScore, worstScore* = **Extract From Solutions** (*solutions, scoresList*) ; // Extract the best, worst, and optimial solutions; and best and worst scores. // Start the updating process using SpaSA equations. // The discoverer location updating process (Equation 1).
4 *i* = 1 ; // Initialize a counter.
5 **while** (*i* ≤ *PD*) **do**
6 **if** (*R*2 < *ST*) **then**
7 $solutions\left[i\right]=solutions\left[i\right]\times exp^{\left(\frac{-t}{\alpha \times T_{max}}\right)}$
8 **else**
9 *solutions*[*i*] = *solutions*[*i*]+*Q*×*L*
10 *i* = *i*+1 ; // Increment the counter. // The followers' location updating process (Equation 2).
11 *i* = 1 ; // Initialize a counter.
12 **while** (*i* ≤ (*N*_*max*_−*PD*)) **do**
13 **if** (*i*>0.5 × *N*_*max*_) **then**
14 $solutions\left[i\right]=Q\times exp^{\left(\frac{worst-solutions\left[i\right]}{i^{2}}\right)}$
15 **else**
16 *solutions*[*i*] = *optimal*+|*solutions*[*i*]−*optimal*| × *A*^+^×*L*
17 *i* = *i*+1 ; // Increment the counter. // The anti-predation behavior (Equation 4).
18 *i* = 1 ; // Initialize a counter.
19 **while** (*i* ≤ *SD*) **do**
20 **if** (*scores*[*i*]≠*bestScore*) **then**
21 *solutions*[*i*] = *best*+β × |*solutions*[*i*]−*best*|
22 **else**
23 $solutions\left[i\right]=solutions\left[i\right]+K\times \left(\frac{\left|solutions\left[i\right]-worst\right|}{\left(scores\left[i\right]-worstScore\right)+\epsilon }\right)$
24 *i* = *i*+1 ; // Increment the counter.
25 **return***solutions* ; // Return the updated population
26 End Function

The steps of the proposed DRDC framework are computed iteratively for a maximum number of iterations *Tmax*. After completing the learning iterations, the optimal combination can be employed in subsequent systems or analyses. The proposed overall parameters learning and hyperparameters optimization technique is summarized by the Algorithm 2.

**Algorithm 2:** Pseudocode for the proposed DRDC framework.

**Input**: *model*, *dataset* (Model name and dataset)
**Output**: *best*, *bestScore* (The best overall score and solution)
1 Partition the dataset into training, testing, and validation portions based on the SR, creating *trainX*, *validationX*, *testX*, *trainY*, *validationY*, and *testY*
2 Create an initial pre-trained TL model, *model*
3 Generate initial solutions, *solutions*
4 Perform the learning SpaSA hyperparameters optimization process for *T*_*max*_ iterations
5 Initialize the iterations counter, *t*, to 1 where *t* ≤ *T*_*max*_
6 **while** (*t* ≤ *T*_*max*_) **do**
7 Initialize the sparrow counter, *i*, to 1 where *i* ≤ *N*_*max*_
8 Initialize the scores list, *scoresList*
9 **while** (*i* ≤ *N*_*max*_) **do**
10 Calculate the fitness score (i.e., accuracy) for the current solution, *score*, using *model*, *solutions*[*i*], *trainX*, *trainY*, *validationX*, and *validationY*
11 Append *score* to *scoresList*
12 Increment the sparrow counter, *i*
13 Update the population using SpaSA (Algorithm 1) with *solutions* and *scoresList*
14 Increment the iterations counter, *t*
15 Return the best score and solution, *best* and *bestScore*

## 5. Experimental results and discussion

### 5.1. The used datasets

The experiments are carried out using two databases. The first dataset is divided into four CT classes, and the second into five histopathology classes. For the first dataset, the authors used **CT KIDNEY DATASET: Normal-Cyst-Tumor and Stone**, which contained 12,446. The second dataset, **kidney cancer**, contained 277 images.

In both datasets, data augmentation is used before the training method to up-sample and normalize the number of images in each category. The first dataset had 20,308 images after equalization, with 5,077 images in each class. In addition, following equalization, the second dataset comprised 355 images, with each class including 67 images. Table 5 provides a brief overview of the parameters of the datasets that were used. Figure 5 shows samples from the used datasets.

**Table 5:** The used datasets specifications summarization.

**Dataset**	**No. of classes**	**Classes**	**No. of images (before)**	**No. of images (after)**
**CT KIDNEY DATASET: Normal-cyst-tumor and stone**	4	“Cyst”, “Normal”, “Stone”, and “Tumor”	12, 446	20, 308
**kidney cancer**	5	“Grade 0”, “Grade 1”, “Grade 2”, “Grade 3”, and “Grade 4”	277	355

### 5.2. Experiments settings

The configurations of different performed experiments are reported in Table 6.

**Table 6:** The common experiments settings.

**Configuration**	**Specifications**
Apply Dataset Shuffling?	Yes (Random)
Input Image Size	(128 × 128 × 3)
Hyperparameters metaheuristic optimizer	Sparrow search algorithm (SpaSA)
Train split ratio	85% to 15% [i.e., 85% for training (and validation) and 15% for testing]
SpaSA size of population	10
SpaSA number of iterations	10
Number of epochs	5
Output activation function	SoftMax
Pre-trained models	VGG19, DenseNet201, MobileNet, VGG16, NASNetMobile, Xception, MobileNetV3Large, and MobileNetV2
Pre-trained parameters initializers	ImageNet
Losses range	Categorical crossentropy, Categorical hinge, KLDivergence, Poisson, Squared Hinge, and Hinge
Parameters optimizers range	Adam, NAdam, AdaGrad, AdaDelta, AdaMax, RMSProp, SGD, Ftrl, SGD Nesterov, RMSProp Centered, and Adam AMSGrad
Dropout range	[0 → 0.6]
Batch size range	4 → 48 (step = 4)
Pre-trained model learn ratio range	1 → 100 (step = 1)
Scaling techniques	Normalize, standard, min max, and max Abs
Apply data augmentation (DA)	[*Yes, No*]
DA rotation range	0° → 45° (step = 1°)
DA width shift range	[0 → 0.25]
DA height shift range	[0 → 0.25]
DA shear range	[0 → 0.25]
DA zoom range	[0 → 0.25]
DA horizontal flip range	[*Yes, No*]
DA vertical flip range	[*Yes, No*]
DA brightness range	[0.5 → 2.0]
Scripting language	Python
Python major packages	Tensorflow, Keras, NumPy, OpenCV, SciPy, and Matplotlib
Working environment	Google Colab with GPU [i.e., Intel(R) Xeon(R) CPU @ 2.00GHz, Tesla T4 16 GB GPU, CUDA v.11.2, and 12 GB RAM]

### 5.3. The Four-classes dataset experiment

The settings for the four-classes dataset are depicted in Table 7.

**Table 7:** Four-classes specific experiments configurations.

**Configuration**	**Specifications**
Dataset Sources	https://www.kaggle.com/nazmul0087/ct-kidney-dataset-normal-cyst-tumor-and-stone
Number of Classes	4
Classes	“Cyst”, “Normal”, “Stone”, and “Tumor”
Dataset Size before Data Balancing	“Cyst”: 3, 709, “Normal”: 5, 077, “Stone”: 1, 377, and “Tumor”: 2, 283
Dataset Size after Data Balancing	“Cyst”: 5, 077, “Normal”: 5, 077, “Stone”: 5, 077, and “Tumor”: 5, 077

The TP, TN, FP, and FN of the best solutions after each pre-trained model's learning and optimization operations on the Four-classes dataset are reported in Table 8. The DenseNet201 pre-trained model recorded the lowest FP and FN values. MobileNetV3Large recorded the greatest FP and FN values.

**Table 8:** CM findings for the Four-classes dataset.

**Model name**	**TP**	**TN**	**FP**	**FN**
VGG16	19,920	60,578	334	384
VGG19	20,073	60,676	224	227
Xception	20,292	60,900	12	12
DenseNet201	20,300	60,910	2	4
MobileNet	20,300	60,908	4	4
MobileNetV2	20,067	60,739	173	237
MobileNetV3 Large	19,200	59,913	999	1,104
NASNetMobile	20,134	60,770	82	150

Table 9 displays the best solution combinations following each model's learning and optimization process (LOP). Four models recommend the KLDivergence loss, while two models only suggest Categorical Crossentropy and Poisson. Three models recommend the SGD Nesterov parameters optimizer, while two only recommend AdaGrad and AdaMax. Three models recommend the standardization and max-abs scaler. Finally, five models recommended applying data augmentation.

**Table 9:** The best solutions following the LOP for the Four-class dataset.

**Model name**	**Loss**	**Batch size**	**Dropout**	**TF learn ratio**	**Optimizer**	**Scaler**	**Apply augmentation**	**Rotation range**	**Width shift range**	**Height shift range**	**Shear range**	**Zoom range**	**Horizontal flip**	**Vertical flip**	**Brightness range**
VGG16	Poisson	24	0.01	22	SGD Nesterov	MinMax	No	N/A	N/A	N/A	N/A	N/A	N/A	N/A	N/A
VGG19	Categorical Hinge	20	0.16	89	AdaGrad	MaxAbs	Yes	44	0.14	0.14	0.05	0.21	Yes	No	1.25-1.85
Xception	KLDivergence	24	0.46	89	AdaMax	Standardization	No	N/A	N/A	N/A	N/A	N/A	N/A	N/A	N/A
DenseNet201	KLDivergence	12	0.18	52	AdaGrad	Standardization	Yes	3	0.22	0.14	0.04	0.03	No	Yes	1.01-1.86
MobileNet	Poisson	24	0.22	46	AdaMax	Normalization	Yes	38	0.07	0.08	0.12	0.03	Yes	No	1.35-1.89
MobileNetV2	KLDivergence	16	0.58	76	SGD Nesterov	Standardization	Yes	35	0.11	0.09	0.17	0.11	No	Yes	0.61-1.81
MobileNetV3 Large	Categorical Hinge	16	0.27	42	SGD Nesterov	MaxAbs	No	N/A	N/A	N/A	N/A	N/A	N/A	N/A	N/A
NASNetMobile	KLDivergence	44	0.33	41	SGD	MaxAbs	Yes	0	0.1	0.08	0.03	0.12	Yes	No	1.23-1.24

We can present several performance measures based on the data reported in Table 8 and the learning history. The measurements reported are classified into two groups. The first reflects the metrics that must be optimized (Table 10). In the second, we see the metrics that need to be reduced (Table 11).

**Table 10:** The Four-classes dataset evaluates the maximized metrics.

**Model name**	**Accuracy (%)**	**F1 (%)**	**Precision (%)**	**Sensitivity (%)**	**Specificity (%)**	**AUC (%)**	**IoU (%)**	**Cosine similarity (%)**
VGG16	98.28	98.22	98.35	98.11	99.45	99.91	94.78	98.21
VGG19	98.89	98.89	98.90	98.88	99.63	99.61	99.17	98.96
Xception	99.94	99.94	99.94	99.94	99.98	99.98	99.71	99.94
DenseNet201	99.98	99.98	99.99	99.98	100	100	99.61	99.98
MobileNet	99.98	99.98	99.98	99.98	99.99	100	99.79	99.97
MobileNetV2	99.05	98.97	99.14	98.83	99.72	99.98	97.43	99.08
MobileNetV3Large	94.83	94.78	95.04	94.56	98.36	99.23	94.29	95.56
NASNetMobile	99.50	99.42	99.59	99.26	99.87	99.99	97.24	99.37

**Table 11:** The Four-classes dataset evaluates the minimized metrics.

**Model name**	**Categorical cross- entropy**	**Kullback Leibler divergence**	**Categorical Hinge**	**Hinge**	**Squared Hinge**	**Poisson**	**Logcosh error**	**Mean absolute error**	**Mean IoU**	**Mean squared error**	**Mean squared logarithmic error**	**Root mean squared error**
VGG16	0.079	0.079	0.112	0.780	0.789	0.270	0.004	0.030	0.375	0.009	0.004	0.094
VGG19	0.075	0.074	0.023	0.756	0.761	0.268	0.002	0.006	0.763	0.005	0.002	0.071
Xception	0.005	0.005	0.006	0.752	0.752	0.251	0.000	0.002	0.377	0.000	0.000	0.018
DenseNet201	0.004	0.004	0.007	0.752	0.752	0.251	0.000	0.002	0.381	0.000	0.000	0.011
MobileNet	0.003	0.003	0.004	0.751	0.751	0.251	0.000	0.001	0.403	0.000	0.000	0.013
MobileNetV2	0.040	0.040	0.055	0.765	0.769	0.260	0.002	0.015	0.514	0.004	0.002	0.067
MobileNetV3 Large	0.174	0.174	0.140	0.786	0.807	0.293	0.009	0.036	0.392	0.020	0.010	0.143
NASNetMobile	0.037	0.037	0.055	0.765	0.769	0.259	0.002	0.015	0.375	0.003	0.002	0.057

We can claim that the DenseNet201 and MobileNet pre-trained models perform the best for the Four-classes dataset.

### 5.4. The Five-classes dataset experiment

The experiment settings for the Five-classes dataset are summarized in Table 12.

**Table 12:** Five-classes specific experiments configurations.

**Configuration**	**Specifications**
Dataset Source	https://www.kaggle.com/atreyamajumdar/kidney-cancer
Number of Classes	5
Classes	“Grade 0”, “Grade 1”, “Grade 2”, “Grade 3”, and “Grade 4”
Dataset Size before Data Balancing	“Grade 0”: 45, “Grade 1”: 45, “Grade 2”: 60, “Grade 3”: 67, and “Grade 4”: 60
Dataset Size after Data Balancing	“Grade 0”: 67, “Grade 1”: 67, “Grade 2”: 67, “Grade 3”: 67, and “Grade 4”: 67

The TP, TN, FP, and FN of the best solutions for each pre-trained model after learning and optimization operations for the Four-classes dataset are reported in Table 13. The pre-trained DenseNet201 model, for example, has the lowest FP and FN values. In contrast, MobileNetV3Large has the highest FP and FN values.

**Table 13:** The Five-classes dataset CM results.

**Model Name**	**TP**	**TN**	**FP**	**FN**
VGG16	229	1,287	25	99
VGG19	313	1,306	6	15
Xception	312	1,248	0	0
DenseNet201	320	1,280	0	0
MobileNet	323	1,308	4	5
MobileNetV2	318	1,327	1	14
MobileNetV3Large	91	1,320	8	241
NASNetMobile	230	1,327	1	102

Table 14 displays the best solution combinations for each model. It demonstrates that three models recommend the Poisson and Categorical Crossentropy losses. Four models recommend the min-max and normalization scalers. Four models recommend the Adam optimizer. Finally, all models suggest using data augmentation.

**Table 14:** After the LOP, the best solutions for the Five-classes dataset.

**Model name**	**Loss**	**Batch size**	**Dropout**	**TF learn ratio**	**Optimizer**	**Scaler**	**Apply augmentation**	**Rotation range**	**Width shift range**	**Height shift range**	**Shear range**	**Zoom range**	**Horizontal flip**	**Vertical flip**	**Brightness range**
VGG16	Squared Hinge	8	0.51	68	SGD Nesterov	MinMax	Yes	5	0.11	0.15	0.22	0.2	No	No	0.57-1.37
VGG19	Poisson	8	0.25	13	Adam	MinMax	Yes	23	0.17	0.11	0.14	0.09	Yes	Yes	0.58–1.24
Xception	Poisson	24	0.17	70	AdaMax	Normalization	Yes	36	0.17	0.01	0.03	0.04	No	No	1.06–1.41
DenseNet201	Poisson	16	0.56	7	RMSProp	MinMax	Yes	5	0.17	0.16	0.25	0.09	No	Yes	0.84–1.04
MobileNet	Categorical Hinge	8	0.44	12	SGD	MinMax	Yes	29	0.02	0.19	0.18	0.13	No	Yes	1.09–1.73
MobileNetV2	Categorical Crossentropy	4	0	0	Adam	Normalization	Yes	0	0	0	0	0	Yes	Yes	0.5–0.5
MobileNetV3 Large	Categorical Crossentropy	4	0	0	Adam	Normalization	Yes	7	0.03	0	0	0	Yes	Yes	0.5–0.5
NASNetMobile	Categorical Crossentropy	4	0	0	Adam	Normalization	Yes	0	0	0	0	0	Yes	Yes	0.5–0.5

Several performance indicators based on the values are in Table 13. The measurements reported are classified into two groups. The first identifies the metrics that must be optimized (i.e., Accuracy, F1, Precision, Sensitivity, Recall, Specificity, AUC, IoU, and Cosine Similarity). The second category reflects the metrics that must be reduced (i.e., Categorical Crossentropy, Kullback Leibler Divergence, Categorical Hinge, Hinge, Squared Hinge, Poisson, Logcosh Error, Mean Absolute Error, Mean Squared Error, Mean Squared Logarithmic Error, and Root Mean Squared Error). Table 15 reports the first category metrics, while Table 16 reports the second category.

**Table 15:** The Five-classes dataset experiments with the maxmimized metrics.

**Model name**	**Accuracy (%)**	**F1 (%)**	**Precision (%)**	**Sensitivity (%)**	**Specificity (%)**	**AUC (%)**	**IoU (%)**	**Cosine similarity (%)**
VGG16	81.10	76.21	90.91	69.82	98.09	97.45	75.84	85.08
VGG19	97.56	96.53	98.17	95.43	99.54	99.82	87.18	95.65
Xception	100	100	100	100	100	100	98.58	99.86
DenseNet201	100	100	100	100	100	100	99.74	99.99
MobileNet	98.78	98.62	98.78	98.48	99.70	99.97	96.99	98.45
MobileNetV2	98.19	96.88	99.70	95.78	99.92	99.96	85.50	96.10
MobileNetV3 Large	62.65	32.36	43.67	27.41	99.40	88.34	56.91	67.17
NASNetMobile	95.78	74.71	88.86	69.28	99.92	99.61	71.98	88.87

**Table 16:** The Five-classes dataset experiments with the minimized metrics.

**Model name**	**Categorical cross entropy**	**Kullback Leibler divergence**	**Cate gorical Hinge**	**Hinge**	**Squared Hinge**	**Poisson**	**Log cosh error**	**Mean absolute error**	**Mean IoU**	**Mean squared error**	**Mean squared logarithmic error**	**Root mean squared error**
VGG16	0.489	0.489	0.503	0.923	0.976	0.298	0.025	0.123	0.400	0.053	0.025	0.230
VGG19	0.193	0.193	0.269	0.860	0.878	0.239	0.009	0.060	0.400	0.018	0.009	0.135
DenseNet201	0.003	0.003	0.005	0.801	0.801	0.201	0.000	0.001	0.462	0.000	0.000	0.007
Xception	0.016	0.016	0.028	0.806	0.807	0.203	0.000	0.006	0.475	0.001	0.000	0.028
MobileNet	0.053	0.053	0.067	0.814	0.820	0.211	0.003	0.014	0.414	0.006	0.003	0.076
MobileNetV2	0.210	0.210	0.282	0.867	0.885	0.242	0.009	0.067	0.400	0.018	0.009	0.134
MobileNetV3 Large	0.996	0.996	0.875	1.028	1.133	0.399	0.049	0.228	0.400	0.105	0.051	0.324
NASNetMobile	0.494	0.494	0.512	0.939	0.986	0.299	0.022	0.139	0.400	0.047	0.022	0.216

**Figure 6:**
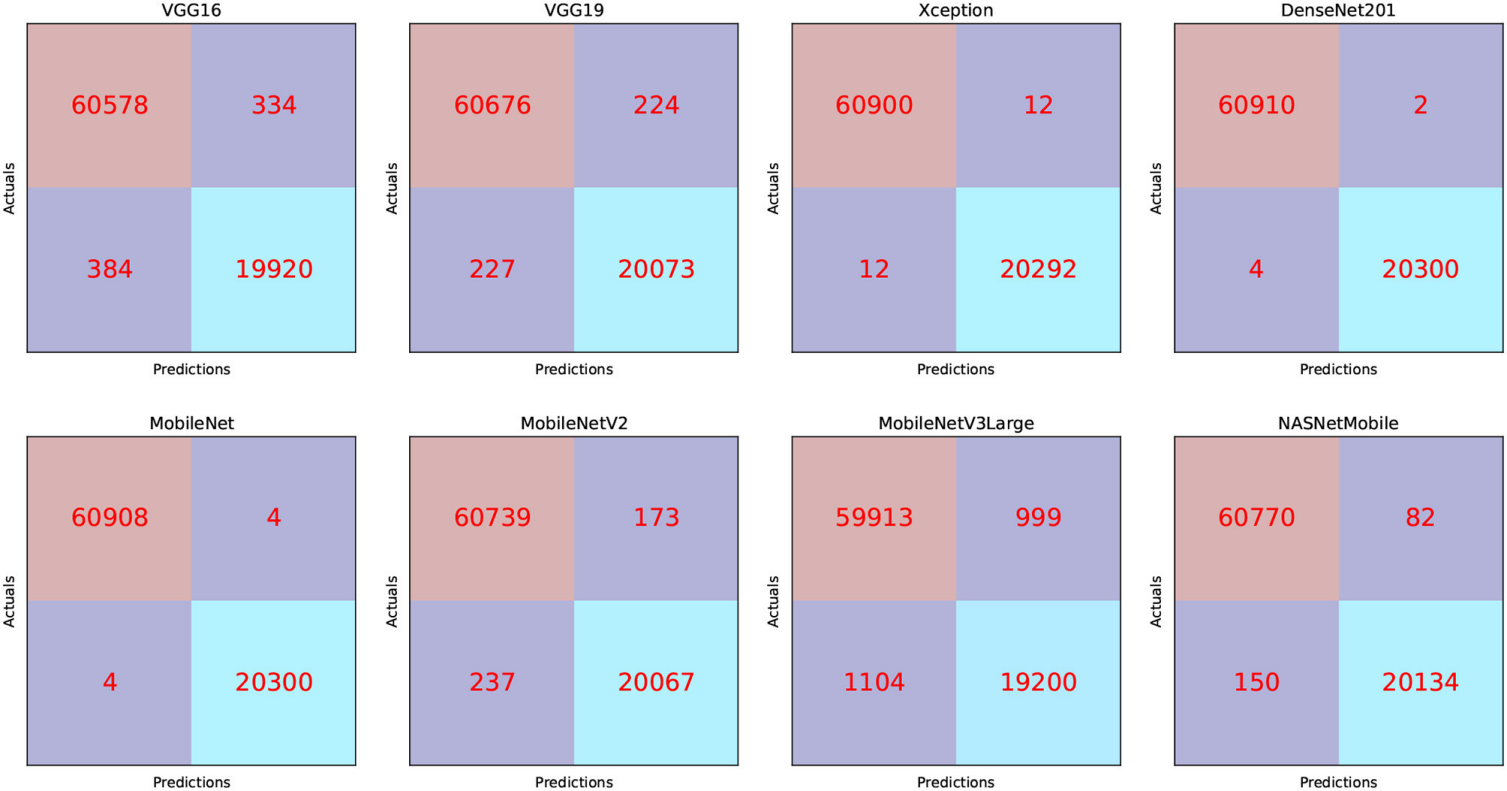
CM related to the Four-classes dataset.

**Figure 7:**
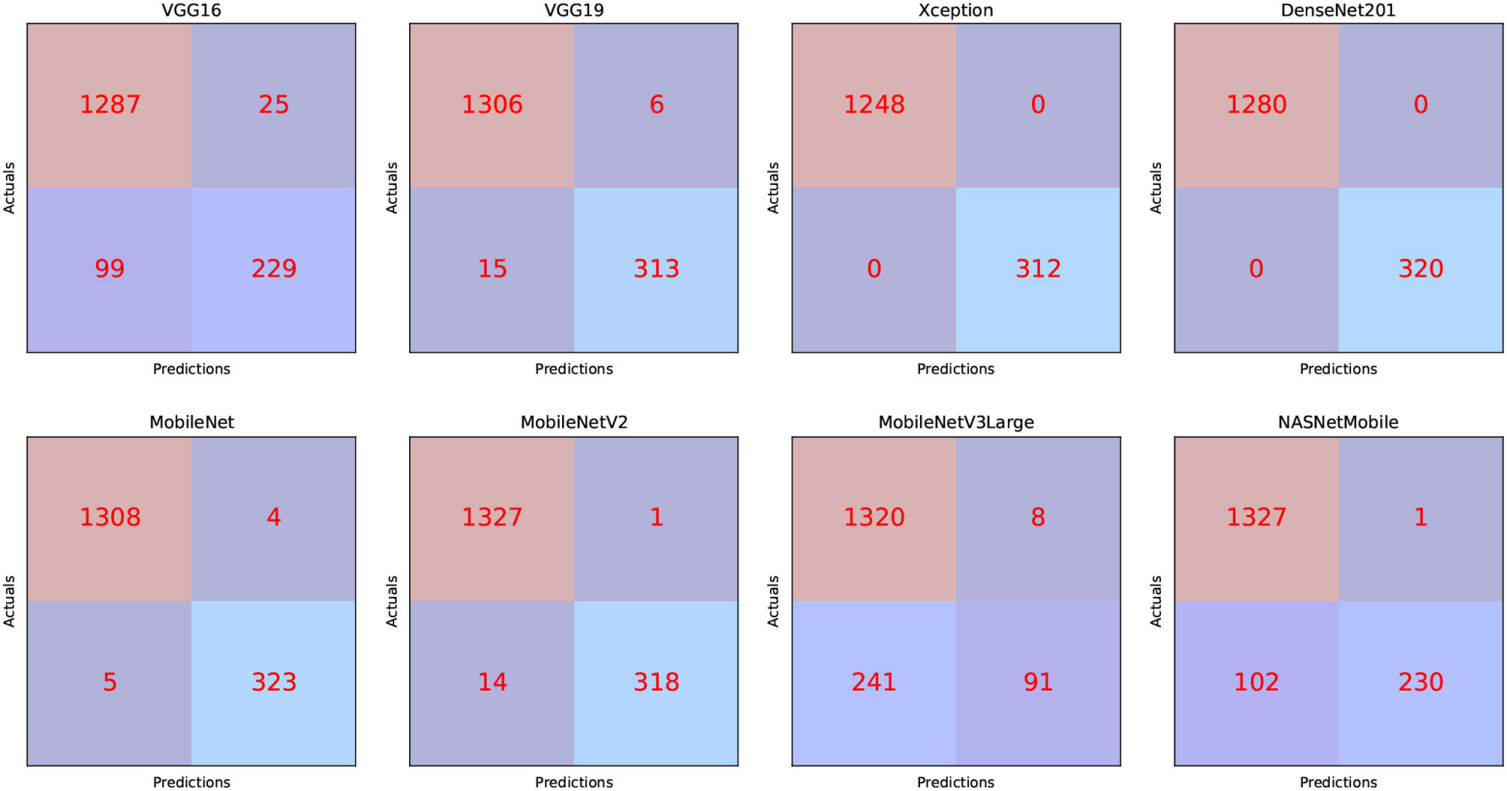
CM related to the Five-classes dataset.

We can infer that the DenseNet201 and Xception pre-trained models are the best concerning the second dataset. The graphical confusion matrices (CM) constructed using the Four-classes, and Five-classes datasets are shown in Figures 6, 7.

**Figure 8:**
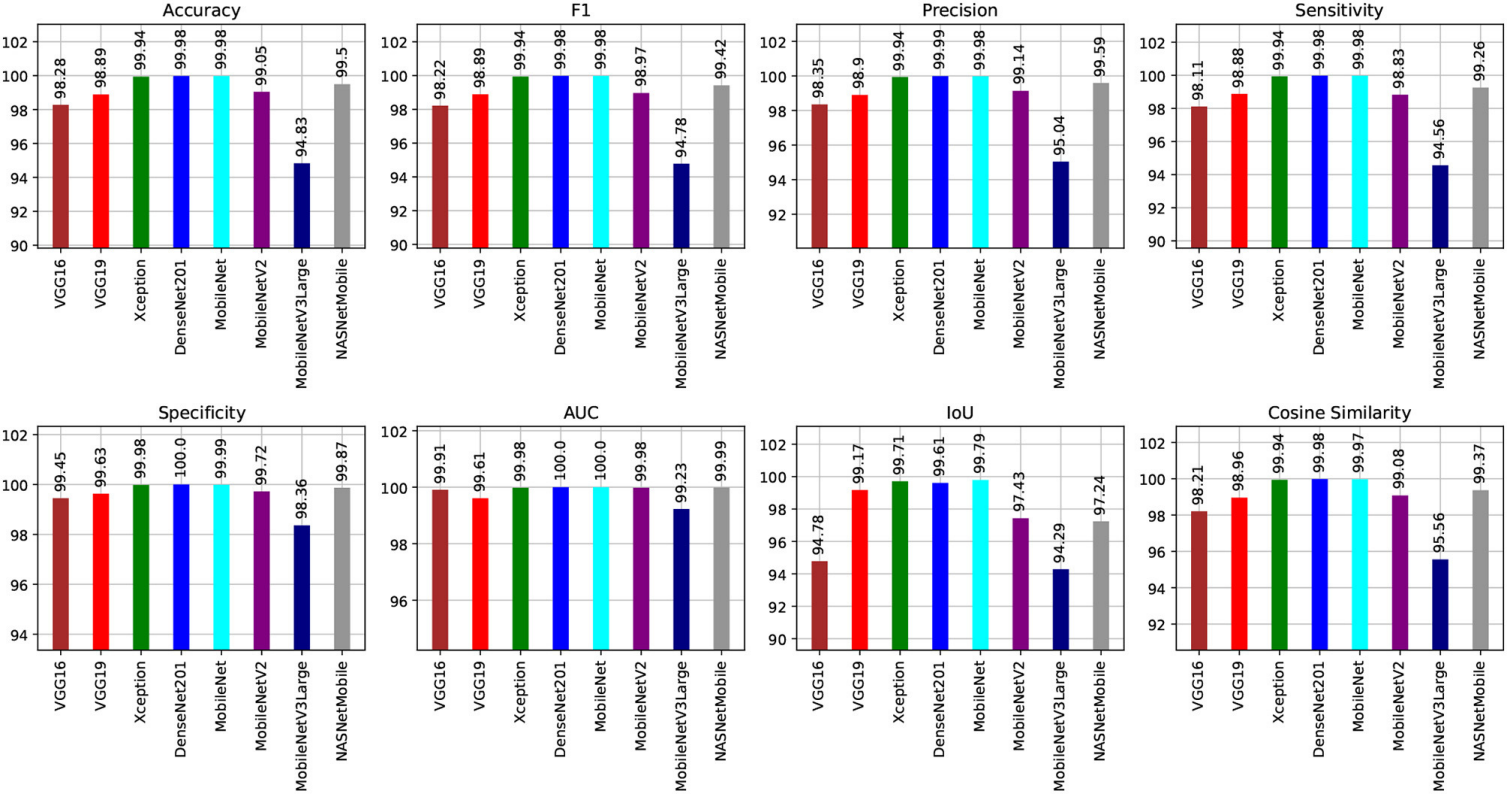
Summarization of the learning and optimization experiments related to the Four-classes dataset.

**Figure 9:**
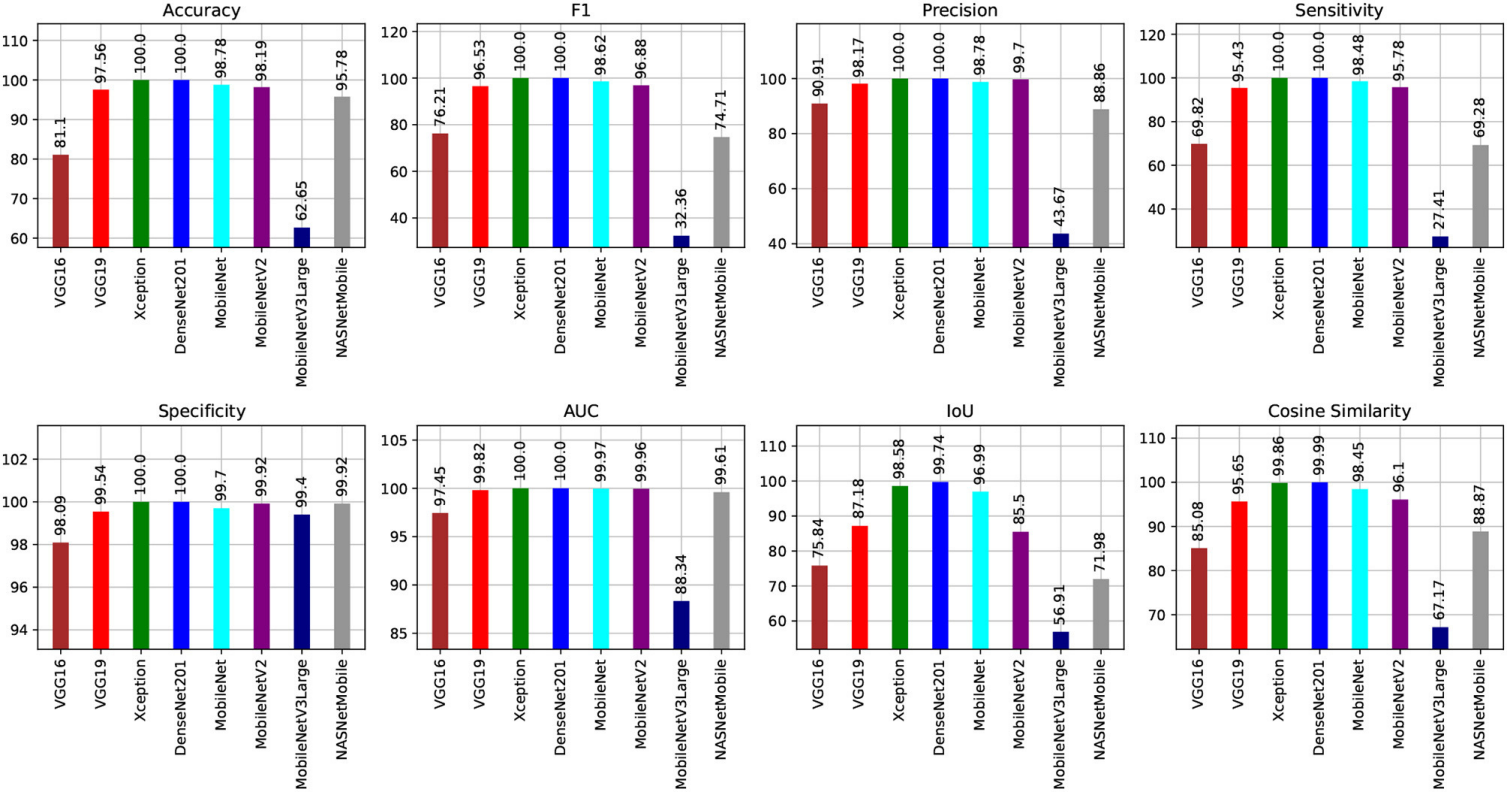
Summarization of the learning and optimization experiments related to the Five-classes dataset.

Figures 8, 9 show graphical summaries of the learning process outcomes for the two datasets.

Table 17 compares the proposed framework to relevant studies. It demonstrates that the DRDC framework outperforms the framework presented by Yildirim et al. (3).

**Table 17:** A comparison of the proposed framework and related studies.

**Study**	**Year**	**Dataset**	**Approach**	**Best accuracy**
Yildirim et al. (3)	2021	2021 Coronal CT	DL	96.82%
The DRDC Framework	2022	CT	Hybrid (SpaSA and TL)	99.98%
The DRDC Framework	2022	Histopathological	Hybrid (SpaSA and TL)	100%

### 5.5. Misclassified images analysis

**Figure 10:**
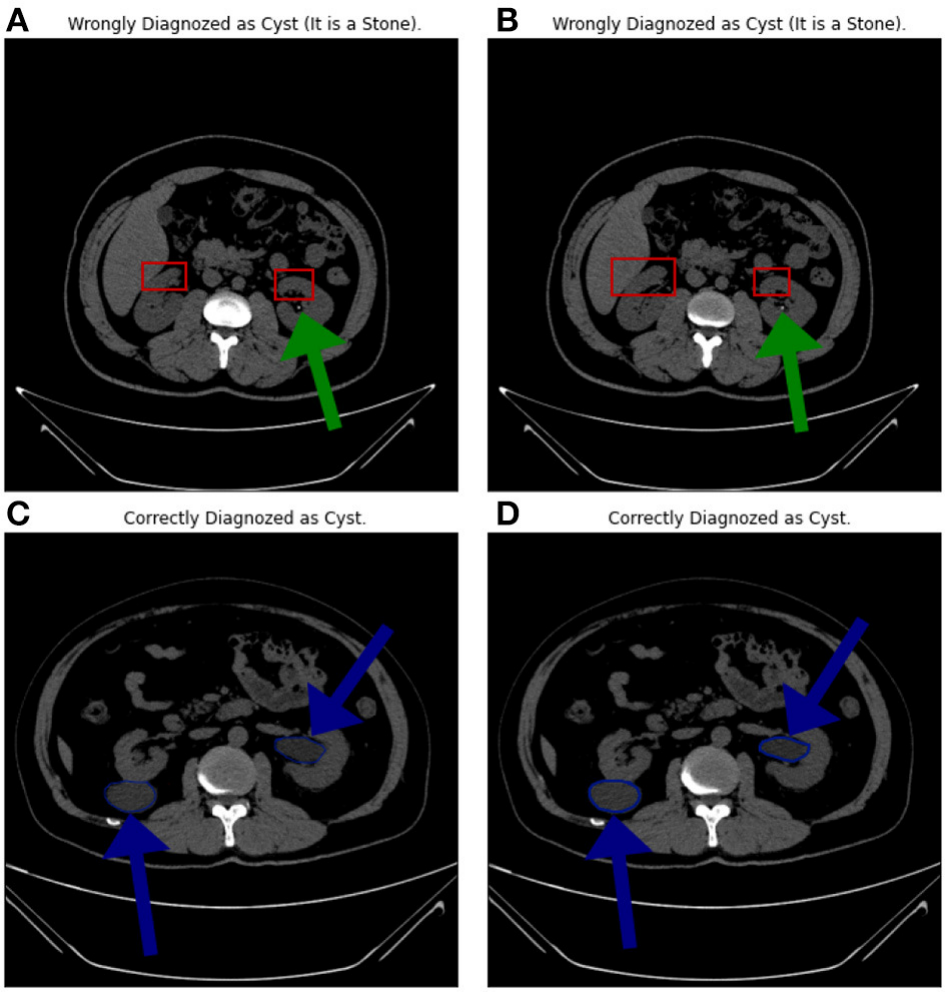
**(A–D)** Samples from the misclassified images.

Figure 10 shows four samples where the upper two samples are diagnosed incorrectly while the lower two are diagnosed correctly. The upper two samples are from the “Stone” category while diagnosed as “Cyst”. The lower two samples are from the “Cyst” category. The green arrows show the locations of the stones. The blue arrows show the cyst locations. The authors think that the reasons behind the misclassification can be (1) the small size of the stones, (2) the size of the kidney itself, and (3) the common portion in the scans, which is represented by the red rectangles.

## 6. Conclusions and future work

A new AI-powered transfer learning framework has been proposed for detecting renal diseases at an early stage, which could potentially transform the way medical professionals diagnose and treat these conditions. Renal diseases, including kidney stones and renal cancer, are a widespread health issue globally, and timely detection is crucial to effectively treat and prevent chronic kidney disease.

The application of deep learning techniques, like convolutional neural networks and pre-trained models, can significantly improve the accuracy and reliability of renal disease diagnosis. Pre-trained CNN models are particularly helpful when working with a limited dataset, and fine-tuning their hyperparameters can further boost their performance.

To optimize the performance of pre-trained models, the study utilized the Sparrow search algorithm (SpaSA) to identify the best models for the four-class and five-class datasets. The DenseNet201 and MobileNet pre-trained models were the most effective for the four-class dataset, while the DenseNet201 and Xception pre-trained models were the best for the five-class dataset. The study recommends using the KLDivergence loss and the SGD Nesterov parameters optimizer for the four-class dataset.

The study also performed manual error analysis to enhance the pre-trained models' performance, which could lead to more precise diagnoses and better treatment options for patients with renal diseases.

The proposed framework can be further improved by applying various metaheuristics to tune the classifier and optimizer parameters. In the future, combining classifiers and optimization for smartphone deployment can make this technology more accessible to medical professionals and patients.

Overall, the proposed AI-based transfer learning framework for the early and accurate detection of renal diseases has the potential to greatly enhance the accuracy and reliability of diagnosis and treatment. The study's findings suggest that the proposed framework outperforms other state-of-the-art classification models, and future research can further improve its performance and accessibility.

## Data availability statement

The original contributions presented in the study are included in the article/supplementary material, further inquiries can be directed to the corresponding author.

## Author contributions

All authors listed have made a substantial, direct, and intellectual contribution to the work and approved it for publication.
